# Fate of the capping agent of biologically produced gold nanoparticles and adsorption of enzymes onto their surface

**DOI:** 10.1038/s41598-023-31792-5

**Published:** 2023-03-25

**Authors:** Parastoo Pourali, Volha Dzmitruk, Miroslav Pátek, Eva Neuhöferová, Milan Svoboda, Veronika Benson

**Affiliations:** 1 Institute of Microbiology, Czech Academy of Sciences, 142 20 Prague, Czech Republic; 2 Center of Molecular Structure, Institute of Biotechnology, Czech Academy of Sciences, 252 50 Prague, Czech Republic; 3 Institute of Analytical Chemistry, Czech Academy of Sciences, 602 00 Brno, Czech Republic

**Keywords:** Biological techniques, Biotechnology, Microbiology

## Abstract

Enzymotherapy based on DNase I or RNase A has often been suggested as an optional strategy for cancer treatment. The efficacy of such procedures is limited e.g. by a short half-time of the enzymes or a low rate of their internalization. The use of nanoparticles, such as gold nanoparticles (AuNPs), helps to overcome these limits. Specifically, biologically produced AuNPs represent an interesting variant here due to naturally occurring capping agents (CA) on their surface. The composition of the CA depends on the producing microorganism. CAs are responsible for the stabilization of the nanoparticles, and promote the direct linking of targeting and therapeutic molecules. This study provided proof of enzyme adsorption onto gold nanoparticles and digestion efficacy of AuNPs-adsorbed enzymes. We employed *Fusarium oxysporum* extract to produce AuNPs. These nanoparticles were round or polygonal with a size of about 5 nm, negative surface charge of about − 33 mV, and maximum absorption peak at 530 nm. After the adsorption of DNAse I, RNase A, or Proteinase K onto the AuNPs surface, the nanoparticles exhibited shifts in surface charge (values between − 22 and − 13 mV) and maximum absorption peak (values between 513 and 534 nm). The ability of AuNP-enzyme complexes to digest different targets was compared to enzymes alone. We found a remarkable degradation of ssDNA, and dsDNA by AuNP-DNAse I, and a modest degradation of ssRNA by AuNP-RNase A. The presence of particular enzymes on the AuNP surface was proved by liquid chromatography–mass spectrometry (LC–MS). Using SDS-PAGE electrophoresis, we detected a remarkable digestion of collagen type I and fibrinogen by AuNP-proteinase K complexes. We concluded that the biologically produced AuNPs directly bound DNase I, RNase A, and proteinase K while preserving their ability to digest specific targets. Therefore, according to our results, AuNPs can be used as effective enzyme carriers and the AuNP-enzyme conjugates can be effective tools for enzymotherapy.

## Introduction

Protein-based treatment in curing pathologies such as inflammation, diabetes, cancer, and degenerative diseases has attracted more attention in recent years^1,2^. The therapeutic protein agents range from small antibodies, growth factors, and cytokines to various enzymes. They usually exhibit high specificity and can be therefore used for targeted therapies. Of the enzymes with potential applications in cancer therapy, DNase I and RNase A are used^3^. The sites of application of these enzymes vary. For DNase I, it should be placed outside the cells to degrade extracellular DNA (exDNA), which is located in the neutrophil extracellular traps (NET) along with some proteins such as histone and actin^4^. An increased amount of exDNA and decreased level of DNase I have been previously shown to provide a platform that serves as an extracellular trap for cancer cell attachment, development, and metastasis^4,5^. The subcutaneous injection of pure DNase I in a mouse model led to NET degradation that prevented cancer-associated thrombus formation^6^. In addition, it was reported that the systemic administration (i.e., injection into the tail vein) of a complex of various proteases (e.g., papain, trypsin, and chymotrypsin) and DNase I exhibited better antitumor activity than a single enzyme thanks to the complete destruction of NET^7^. The situation is not straightforward though, since various tumor types produce or trigger the production of proteases that enable tumor metastasis^8^. That complication is outweighed by other molecules such as trypsin^9^, cysteine proteases^10^, pancreatic enzymes^11^, and certain matrix metalloproteinases^12^ that possess protective anticancer effects. Thus, the use of protective proteases or inhibitors of cancer-promoting proteases could still be considered in homeostasis reestablishment and cancer treatment.

When targeting cancer cells, particular attention is paid to RNase A which induces cytotoxic effects after intracellular delivery^13^ into tumor cells (intracellular degradation of tRNA, mRNA, rRNA or non-coding RNAs^14^). Besides direct introduction into cells, the intra-muscular administration of RNase A was also documented to decrease the RNA level in blood^15^. Although the use of the above-mentioned enzymes and other therapeutic protein agents proved to demonstrate some benefits such as low toxicity and high specificity in regulating cellular functions^16^, the short half-life of these enzymes, especially for RNase A, limited the technique. RNase A was shown to be filtered out by the kidney in the first few minutes after injection (half-life < 5 min) which is too short to be effective^17^. Passing through the cell membrane is another obstacle^18^ which can result in poor therapeutic efficacy^19^. The use of specific carriers to deliver these molecules into cells is considered a solution for the above-mentioned issues^20^. Specifically, RNase A was reported as an antineoplastic agent^20^ in conjugates with phenylboronic acid^21^, cationic lipid-based nanoparticles^22^, hyaluronic acid^3^, zeolite nanoparticles^23^, HP nanogels^24^, dextran nanogel^19^, and glyco-gold nanoparticles^25^. The application of DNase I in a complex of DNase I-gold nanoparticles reduced lung metastases in mice^26,27^, since the nanoparticles protected the enzyme from degradation.

Thus nanocarriers seem to be more convenient for the delivery (preferably targeted delivery) of various enzymes into the cells. Of the most biocompatible nanostructures, gold nanoparticles (AuNPs) were found to be most suitable. AuNPs are known to have low toxicity and are mainly used for drug delivery^28,29^. Other AuNP applications include bio-imaging^30^ and photothermal therapy^31^.

AuNPs can be produced by various chemical, physical, and biological methods^32^. Biological techniques provide some advantages, but are not widely used. In the biological approach, various microbial strains or plant extracts provide a bio-reduction of toxic ions. The extra- and/or intracellular materials such as proteins and polysaccharides are responsible for this type of bio-reduction^28,29,33–35^. It was shown that during the reduction process, some microbial substances, which are called *capping agents* surround the surface of the produced NPs^36,37^. These agents often consist of proteins, but their composition varies depending on the type of microorganism used^29^. For example, AuNPs produced extracellularly by the fungus *Fusarium oxysporum*, were found to be composed of two proteins of 25 kDa and 19 kDa by sodium dodecyl sulphate–polyacrylamide gel electrophoresis (SDS-PAGE) analysis^38^. In our previous study with the same fungus, we showed that the capping agents of the extracellularly produced AuNPs were not proteins, but most likely instead small peptides or even amino acids, as documented by the presence of amide I and amide II on the surface of AuNPs detected by Fourier transform infrared spectroscopy^28,29,39^. If the large proteins acted as capping agents, the AuNPs would not be found at the nanoscale. In parallel, we showed that AuNPs could directly conjugate with smaller molecules, such as various antibiotics and drugs^33,39,40^. In summary, biologically produced AuNPs that could bind enzymes or other therapeutic molecules without the addition of toxic chemicals would be good candidates for cancer therapy.

There are several hypothetical situations that should be considered when using biologically produced AuNPs as enzyme carriers. Of these, two hypotheses were of great interest to us 1) the capping agents are digested and enzymes do not bind to AuNPs or 2) enzymes are bound by the AuNPs irrespective of whether the capping agent is degraded or not. We focused on three different enzymes proteinase K, DNase I, and RNase A to address the two hypotheses.

## Results

### Characterization of the produced AuNPs

**Figure 1:**
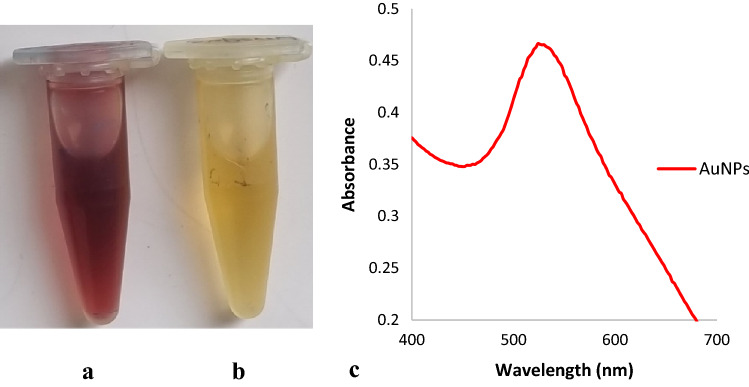
Formation of AuNPs in culture supernatant and their visible absorption spectrum. (**a**) Dispersion containing AuNPs, (**b**) control supernatant without AuNPs, (**c**) visible absorption spectrum of the produced AuNPs, showing maximum absorption peak at 530 nm. The formation of AuNPs can be easily observed by the color change from yellow to crimson.

**Figure 2:**
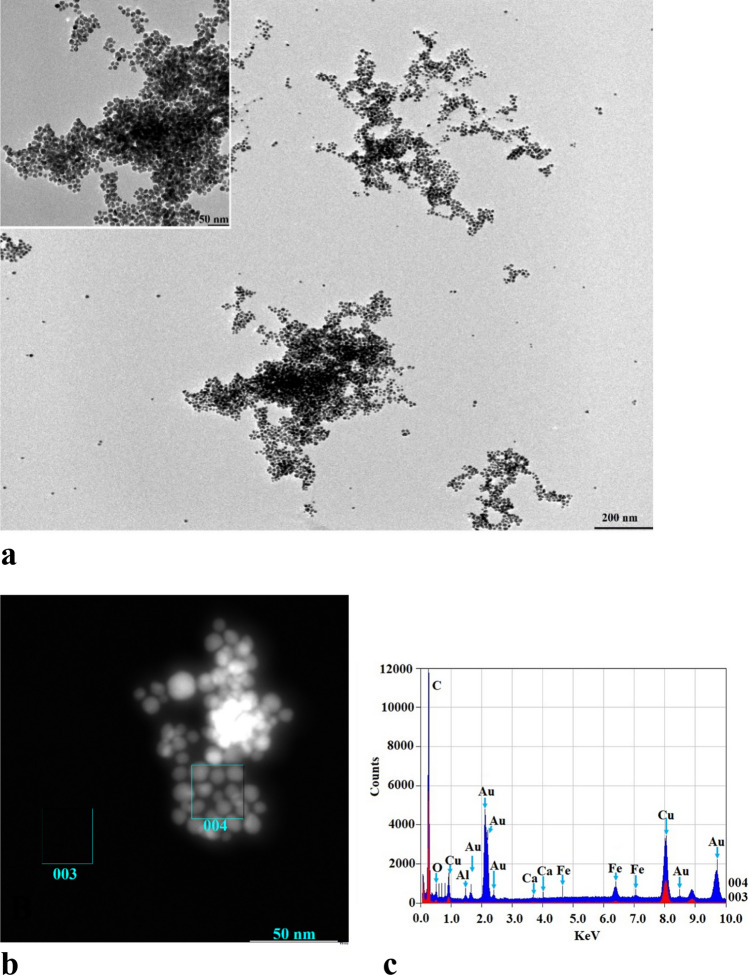
Detection of AuNPs by electron microscopy. (**a**) TEM-obtained image (scale bar = 200 nm and insert photo scale bar = 50 nm), (**b**) representative detail of areas 003 and 004 used for subsequent EDS analysis (scale bar = 50 nm), and (**c**) the EDS spectra overlay of the gated areas with marked blue and red peaks representing individual elements in the areas 004 and 003, respectively.

We produced the AuNPs using the fungus *Fusarium oxysporum* as in our previous study^29^. The formation of AuNPs was recognized according to a color change (from yellow to crimson) of the culture supernatant challenged by HAuCl_4_.3H_2_O. The obtained colors of AuNP-containing dispersion and non-challenged control supernatant can be seen in Fig. 1a,b. Visible spectrophotometer analysis showed that the AuNP dispersion provided a maximum absorption peak at 530 nm (Fig. 1c). Transmission electron microscopy (TEM) showed round or polygonal AuNPs with sizes around 5 nm in the dispersion (Fig. 2b). Energy dispersive X-ray spectroscopy (EDS) confirmed the presence of elemental gold on the surface of TEM copper grids (Fig. 2c). The resulting AuNPs exhibited high negative charge (-33 mV) and small sizes with an average diameter of 4.8 ± 3.1 nm according to the zeta potential and particle size distribution analyses with Zetasizer Ultra (Table 1).

**Table 1:** Size (nm) and zeta potential (mV) of all AuNPs-enzyme samples and AuNPs without any enzyme cargo.

Sample	Zeta potential (mV)		Size (nm)	
	Mean	s.d	Mean	s.d
AuNPs	− 33.29	± 0.22	4.82	± 3.11
AuNPs-RNase A	− 12.92	± 0.71	12.68	± 4.43
AuNPs-DNase I	− 21.53	± 0.84	12.60	± 3.60
AuNPs-proteinase K	− 12.97	± 0.92	10.37	± 4.77

Electron microscopy images proved that the biologically produced AuNPs do not agglomerate even in close contact thanks to their capping agents. The clear separation of individual AuNPs can be seen in the detailed images in Fig. 2a,b.

### Analysis of capping agent by Fourier transform infrared spectroscopy (FTIR)

**Figure 3:**
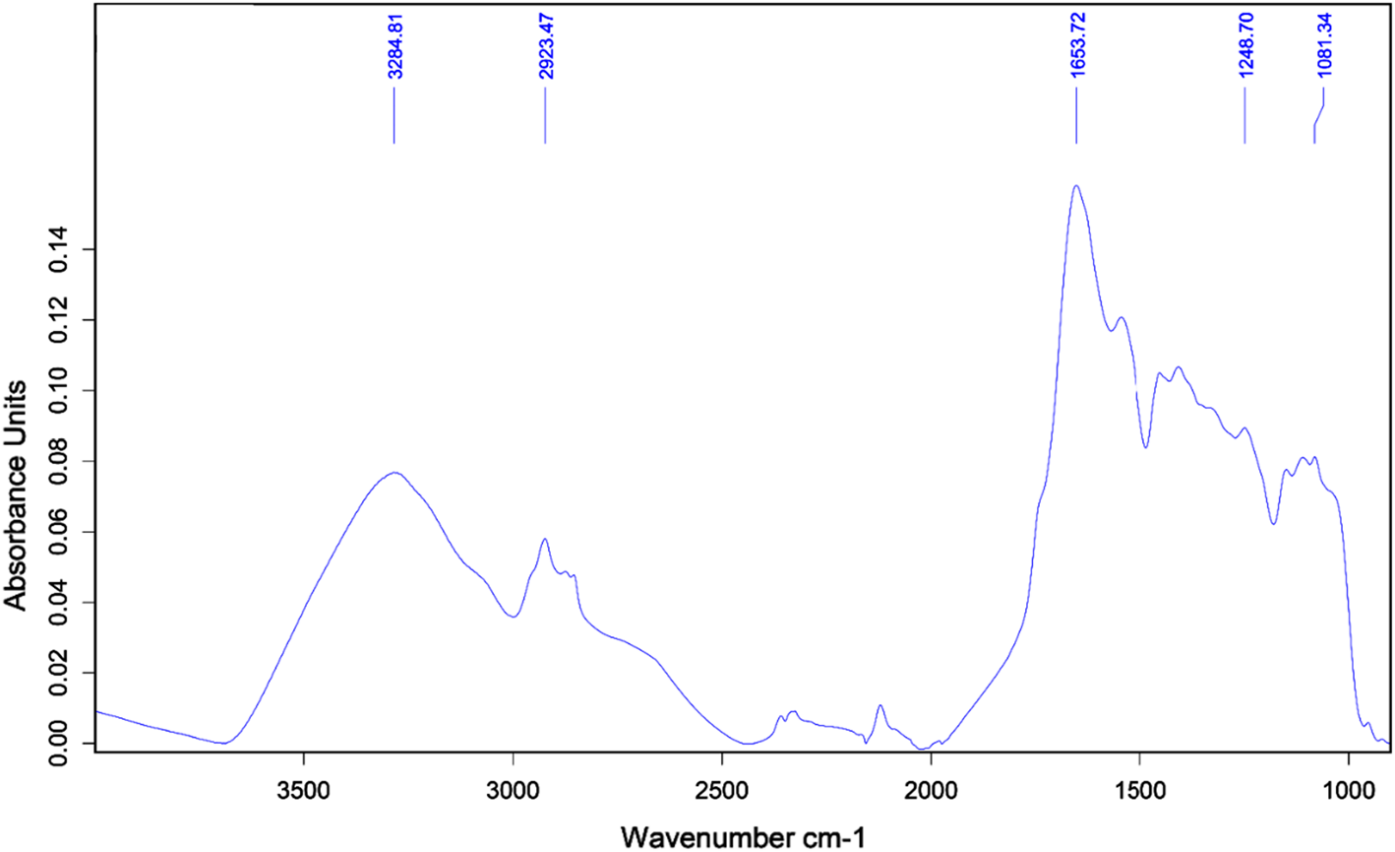
FTIR spectrum of AuNPs with marked positions corresponding to specific chemical bonds. The peak at 3287 cm^−1^ corresponds to N–H stretching and possibly to O–H stretching. The peaks at 2923 cm^−1^, 1653 cm^−1^, 1248 cm^−1^, and 1081 cm^−1^ reflect sp3 C–H, C=C, C–N, and C–O stretching, respectively.

FTIR was used to further analyze the basic composition of capping agents decorating biologically produced AuNPs. The infrared spectrum of AuNPs showed a broad peak centered at 3287 cm^−1^ corresponding to N–H stretching (secondary amine) and possible O–H stretching (carboxylic acid) vibration of AuNPs indicated the presence of peptides or amino acids on the surface of AuNPs (Fig. 3). The peak at 2923 cm^−1^ with nearby subpeaks is the peak position of sp3 C–H stretching (alkane) vibration bands. The peak at 1653 cm^−1^ is the absorption of C=C stretching (alkene) of AuNP, and the peak at 1248 cm^−1^ represents C–N stretching (amine). The peak at 1081 cm^−1^ represents the absorption of C–O stretching (amide) (Fig. 3).

### Assessment of the produced amount of AuNPs using graphite furnace atomic absorption spectroscopy (GF-AAS)

As AuNPs were washed three times, the free Au ions were washed out and GF-AAS could reveal the precise amounts of AuNPs. We measured that under our conditions, the fungus *F. oxysporum* produced 161 ± 20 µg/ml of AuNPs.

### Characterization of AuNPs treated with various enzymes

The main question at the beginning of this study concerned the consequences of enzyme addition to AuNPs and the fate of the enzyme and capping agent. We hypothesized that two possible scenarios may occur on the surface of AuNPs after the application of a digestive enzyme: (1) the capping macromolecules such as nucleic acids or peptides are digested and the enzymes do not bind to the AuNPs or (2) the degrading enzymes are adsorbed onto the AuNPs surface independently of whether the capping agent is degraded or not. We further supposed that the digested matter in case (1) would be released into the supernatant or would remain attached to the particles next to the enzyme.

**Figure 4:**
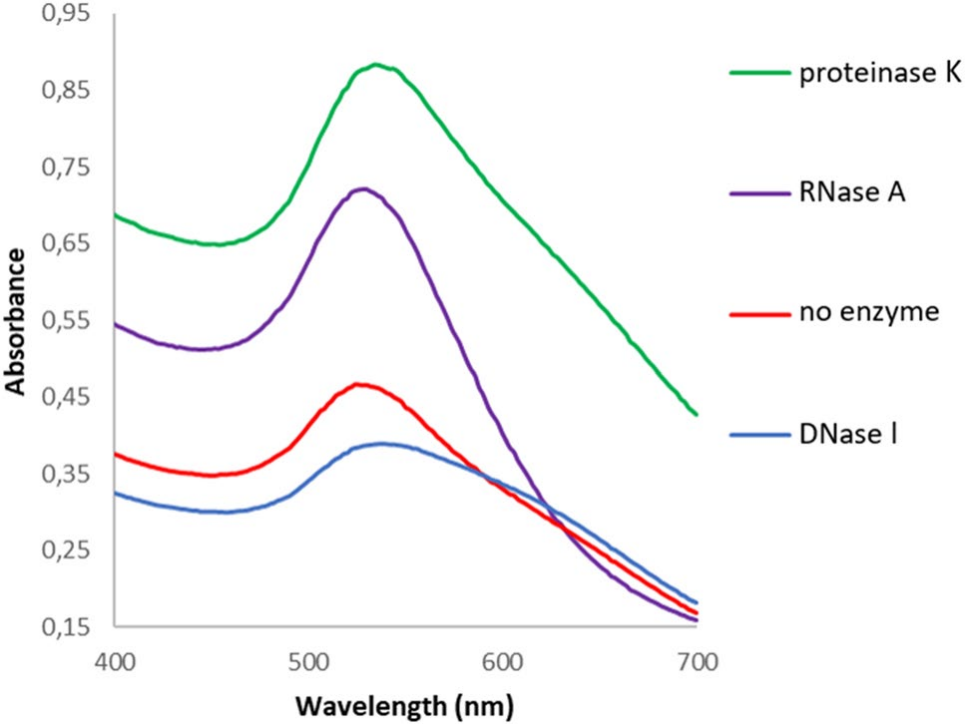
Visible spectra of AuNPs before (no enzyme) and after incubation with various enzymes (i.e., proteinase K, RNase A and DNase I).

After the incubation of AuNPs with various enzymes, the samples were centrifuged and pellets were washed three times before their evaluation by visible spectrophotometry. According to Fig. 4, the maximum absorbance peaks (MAPs) of the washed AuNPs changed after incubation with the enzymes, which is an indication of enzymatic digestion of the capping agents or adsorption of enzymes onto the surface of the AuNPs.

The maxima of the absorption peaks for each AuNPs-enzyme combination differed (Fig. 4). In comparison to AuNPs without cargo, the spectra of AuNPs-DNase I exhibited lower absorbance in its MAP and a broader peak. On the other hand, AuNPs-RNase I and AuNPs-proteinase K exhibited higher absorbance values in their MAP position and the spectra of AuNPs-RNase A showed steeper slopes of the MAP than other samples. The MAPs for enzyme-free AuNPs, AuNPs-DNase I, AuNPs-RNase A, and AuNPs-proteinase K were at 530, 538, 513, and 534 nm, respectively.

Zetasizer analysis showed the changes in the size and zeta potential of AuNPs after incubation with all the enzymes (Table 1).

Table 1 shows that the nanoparticles enlarged and the zeta potential increased after incubation with enzymes. The shifts in AuNP size and net charge suggested that incubations of the AuNPs with the enzymes caused considerable changes on the AuNPs surfaces possibly due to removing the capping agents or adsorption of the enzymes onto the AuNPs. Therefore, further experiments aimed to provide support for our hypotheses.

### Evaluation of the digestion of capping agents and detection of the digestion products

After incubations with DNase I and RNase A, the supernatants of AuNPs-containing samples were tested for the presence of nucleic acids that could be released from the AuNP capping agents by digestion. After the application of DNase I, we detected on average 11.6 ng/µL of free DNA. After the application of RNase A, we detected on average 9.6 ng/µL of free RNA. There were no traces of DNA or RNA in the supernatants of control samples not treated with enzymes.

### Evaluation of the adsorption of the enzymes onto the surface of AuNPs

As was mentioned in Methods, to test the adsorption hypothesis, AuNPs-enzyme complexes were used as test samples and equal amounts of the DNA and RNA substrates were treated with those AuNPs-enzyme complexes. The same pure enzymes served as controls. The starting concentrations of nucleic acids before incubation with AuNPs-enzymes (as test) and enzymes (as control) and their concentrations after the treatment are shown in Table 2.

**Table 2:** Concentrations of ssRNA, ssDNA, and dsDNA in samples before and after incubation with enzymes and AuNPs-enzymes.

Nucleic acid content (ng/µL)	ssRNA	ssDNA	dsDNA
Before incubation with enzymes	1164	548	77
Digestion with enzymes (control)	452	492	53
Digestion with AuNPs-enzymes (test)	585	237	23

We found remarkable reduction (61 and 50%) in ssRNA content after the application of RNase A alone and in a complex with AuNPs, respectively, and both variants exhibited similar digestion efficacy. With ssDNA and dsDNA targets we witnessed a remarkable reduction in the amounts of nucleic acids after the application of enzymes linked to AuNPs (56 and 70%) in contrast to a modest concentration decrease resulting from the enzyme treatment alone (10 and 31%, respectively). It is important to note here that: (1) although the supernatants were recovered with a high-speed centrifuge (22,000×*g*, 30 min), a minor loss of the AuNP fraction should be considered and (2) the AuNPs exhibited a basic absorbance level at a wavelength of 260 nm during the spectrophotometric quantification of the nucleic acids. Thus, we believed that the results obtained by spectrophotometric analysis showed us the trend but not absolute values, and we therefore employed gel electrophoresis as another proof of the digestion of nucleic acids.

### Proof of DNA, RNA, and protein digestion

The presence of nucleic acids and proteins in the samples after treatment with enzymes and AuNP-enzyme complexes, respectively, was further documented by gel electrophoresis.

**Figure 5:**
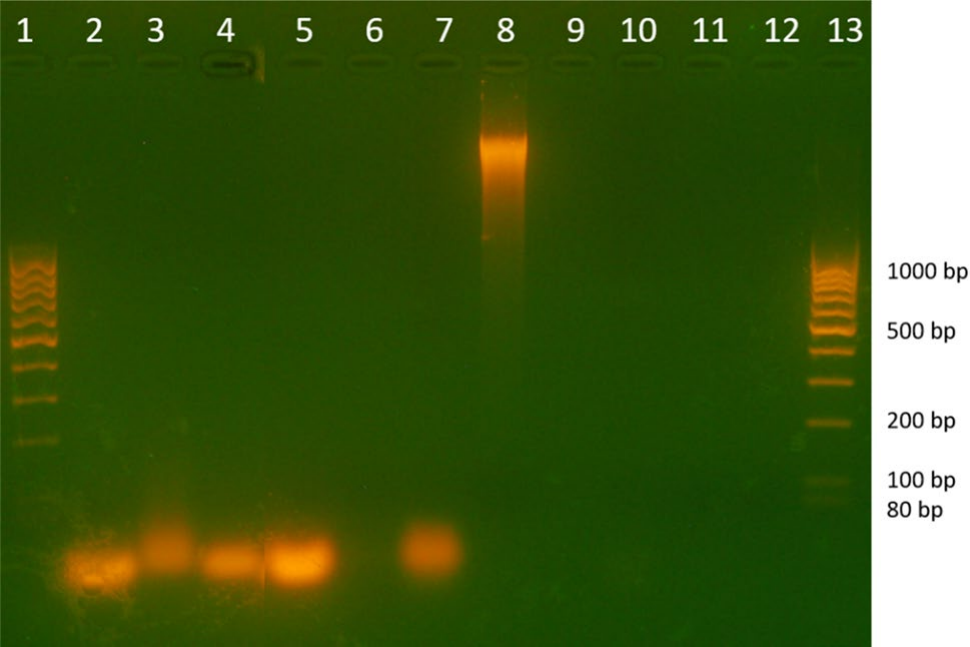
Nucleic acid agarose gel electrophoresis. Lanes (1) and (13) DNA ladder, (2) control ssRNA, (3) ssRNA-RNase A, (4) ssRNA-AuNPs-RNase A, (5) control ssDNA I, (6) ssDNA-DNase I, (7) ssDNA-AuNPs-DNase I, (8) dsDNA, (9) dsDNA-DNase I, (10) dsDNA-AuNPs-DNase I, (11) supernatant from AuNP sample after DNase I application, and (12) supernatant from AuNP sample after RNase A application.

The agarose gel electrophoresis revealed partial or total digestion of the targeted nucleic acid in the samples treated with both enzymes alone and AuNP-enzyme complexes. The presence of sharp bands in the control samples (Fig. 5, lanes 2, 5 & 8) in contrast to the assays (both enzymes and AuNPs-enzymes) reflected enzymatic digestion of the substrates. In some samples, the nucleic acid content was lowered more efficiently when treated with enzymes alone than with the AuNPs-enzymes. Specifically, ssRNA exhibited moderate signs of degradation after treatment with RNase A alone but treatment with AuNP-RNase A resulted in a very mild change if any (Fig. 5, lanes 2–4). ssDNA was effectively degraded by DNase I itself and less effectively by the AuNP-DNase I complex (Fig. 5, lanes 5–7). dsDNA was effectively degraded by both, DNase I alone as well as AuNP-DNase I complex (Fig. 5, lanes 8–10).

To verify the presence of DNase I and RNase A enzymes adsorbed on the surface of the AuNPs, an LC–MS analysis was performed and the data are presented below.

**Figure 6:**
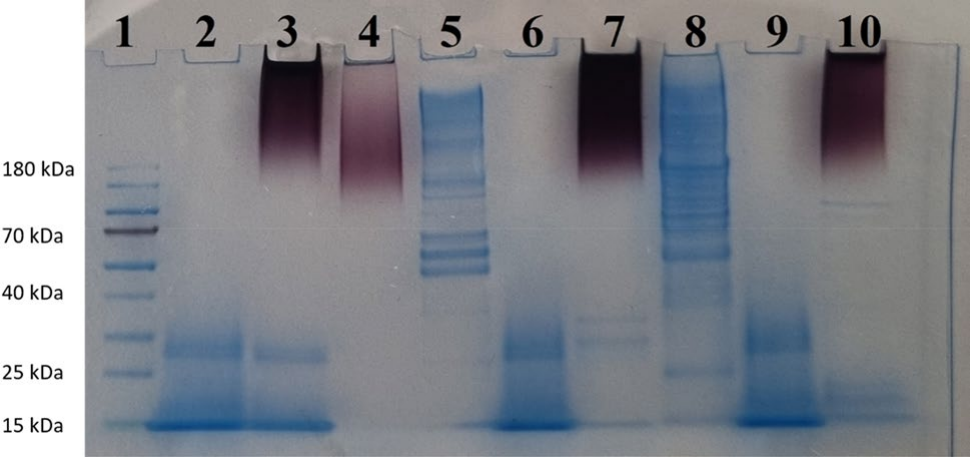
SDS-PAGE showing proteins digested with proteinase K and AuNPs-proteinase K. (1) Pre-stained protein ladder, (2) pure proteinase K, (3) unwashed AuNP-proteinase K, (4) washed AuNP-proteinase K, (5) fibrinogen as control, (6) fibrinogen with proteinase K, (7) fibrinogen with washed AuNP-proteinase K, (8) collagen type I as control, (9) collagen type I with proteinase K, (10) collagen type I with washed AuNP-proteinase K.

Figure 6 shows the SDS-PAGE gel supporting the digestion of protein substrates (fibrinogen and collagen type I) by proteinase K and AuNPs-proteinase K. The pure proteins, proteinase K, collagen type I, and fibrinogen are loaded onto the gel as controls. The presence of the AuNPs in lanes 3, 4, 7, and 10 resulted in the red color of those samples.

As shown by SDS-PAGE (Fig. 6), the band of proteinase K (28.9 kDa^48^) was visible in lane 3 representing unwashed AuNPs-proteinase K samples but it disappeared from the AuNPs-proteinase K sample after three washes (lane 4). As seen in lanes 6 and 9, proteinase K itself digested both fibrinogen and collagen type I, and the protein bands disappeared in comparison with lanes 5 and 8, respectively. This result confirms the activity of the proteinase K. Although the presence of the enzyme could not be detected by SDS-PAGE in the AuNPs-proteinase K sample after washing (lane 4), the results of digestion of fibrinogen and collagen type I by this complex, seen in lanes 7 and 10, are proof that proteinase K was still present on the AuNPs and degraded its substrates. We conclude that a low amount of the enzyme was adsorbed on the surface of AuNPs, and its activity was not suppressed by the adsorption.

### Proof of active nucleases adsorbed on AuNP by LC–MS

**Figure 7:**
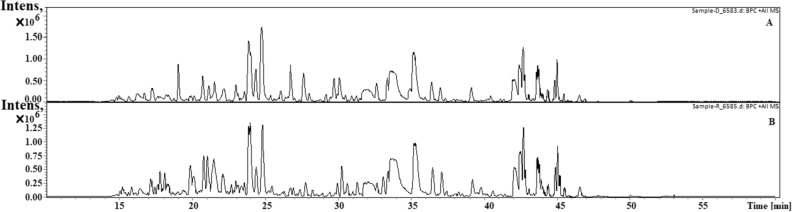
Representative LC–MS chromatogram of AuNPs-DNase I (top) and AuNPs-RNase A (bottom) that served for data mining and detection of the components of AuNP-enzyme complexes presented in Table 3.

From the results presented in the previous sections, it is apparent that the protease bound in the AuNP-proteinase K complex was active. To also prove that the tested nucleases are adsorbed on AuNPs and active, we performed an LC–MS assay for two complexes: AuNPs-DNase I and AuNPs-RNase A. The LC–MS results were matched with the UniProt database and the output list confirmed the presence of the used nucleases in AuNP-DNase I and RNase A samples (Table 3 and Fig. 7).

**Table 3:** Identification of DNase I and RNase molecules after comparison of LC–MS (AuNPs-enzymes) output list with the UniProt database.

Protein group	Protein ID	Accession	Coverage (%)	Avg. mass	Description
2	51	P00639|DNAS1_BOVIN	81	31,346	Deoxyribonuclease-1 OS = Bos taurus OX = 9913 GN = DNASE1 PE = 1 SV = 3
9	19521	P07847|RNAS1_AEPME	73	13,678	Ribonuclease pancreatic OS = Aepyceros melampus OX = 9897 GN = RNASE1 PE = 1 SV = 1
10	19519	P67927|RNAS1_SHEEP	77	13,707	Ribonuclease pancreatic OS = Ovis aries OX = 9940 GN = RNASE1 PE = 1 SV = 1
10	19520	P67926|RNAS1_CAPHI	77	13,707	Ribonuclease pancreatic OS = Capra hircus OX = 9925 GN = RNASE1 PE = 1 SV = 1
4	19518	P61824|RNAS1_BISBI	89	13,690	Ribonuclease pancreatic OS = Bison bison OX = 9901 GN = RNASE1 PE = 1 SV = 1

## Discussion

To reduce the undesirable side effects of protein therapeutics and increase their circulation time and stability, different types of materials are used. Specifically, biologically produced AuNPs are of great interest due to their specific properties. This type of nanoparticle has the exclusive ability to directly bind different types of proteins by dipole–dipole interactions and hydrogen bond formation thanks to the polar capping agents that naturally decorated biological AuNPs^28,33,41^. This phenomenon makes biologically engineered nanoparticles good candidates for protein delivery in the treatment of various diseases, such as cancer.

There were attempts to produce non-biological AuNPs with specific protein-based coatings that facilitated binding to the therapeutic protein molecules, but each of them required additional steps for coating and suffered from limited application due to toxicity, the use of chemical linkers, or lower internalization rate^25,27,42–48^.

Our study shows for the first time the ability of biologically produced AuNPs to directly conjugate to enzymes. The cell membrane is impermeable to proteins^3^ and the NPs carriers accelerate the internalization of enzymes into cancer cells. Thus, the conjugates of different degrading enzymes with the NPs are hypothetically promising candidates for the therapy of pathologies including cancer. There were attempts to target cancer cells with RNase A^21,49^ and DNase I^50^ using lipid-based NPs carriers and cross-linked nanogels (cNG), respectively^51^. In our study, we linked these enzymes directly to biologically produced AuNPs and proved the attachment of the enzymes as well as their activity. The activity of AuNPs-DNase I and AuNPs-RNase A complexes depended on the substrates used. For example, AuNPs-DNase I exhibited the best activity against dsDNA and preferred dsDNA to ssDNA. Some reports on the DNase I-carrying NPs reported the same activity as the pure enzyme^52–54^ and we also observed this phenomenon with ssDNA degradation. However, with dsDNA, the pure DNase I as well as AuNPs-DNase I complex performed with equal efficacy. The digestion of ssDNA in the assays and controls was less effective than the cleavage of dsDNA probably due to considerably lower specific activity of DNase I for ssDNA (500-fold lower) than for dsDNA^55^. The lower activity of DNase I linked to AuNPs resulted from the sensitivity of those molecules to physical denaturation during vortexing that was necessary part of complex preparation (washing steps). As for the efficacy of ssRNA digestion, the surface charge of the complex AuNP-RNase A was about − 13 mV, which points towards lower stability of the complex and likely contributes to its lower efficacy. We did not include dsRNA in this study because RNase A is an ssRNA-specific endonuclease that has a strong preference for ssRNA over dsRNA^56^. Therefore, the choice of substrate and the best corresponding enzyme should be tested and considered in future studies.

The poor digestion of ssRNA by AuNPs-RNase A can be also explained by the adsorption of the ssRNA onto AuNPs. It was shown that the non-biologically prepared AuNPs functionalized with thiol-containing polyethylene glycol poly(2-N,N-dimethylamino)ethyl methacrylate (SH-PEG-PAMA) attached siRNA molecules and prevented their enzymatic degradation^57,58^. After the contact of AuNPs with ssRNA molecules, the ssRNAs adsorb to the AuNP surface next to the enzyme (at various distances), and their degradation would be reduced by a spatial barrier. Further analysis is needed to confirm this hypothesis in the future. If the adsorption of substrate nucleic acids onto AuNPs remarkably protected them from their enzymatic degradation, we would need to coat the surface of AuNPs-enzymes with specific agents to avoid that secondary adsorption.

One of the goals while using nanoparticles for enzymotherapy is to use the lowest amounts of enzymes that are still effective. Because AuNPs are small (approximately 5 nm in diameter), we did not expect the entire AuNP surface to be covered with an enzyme that is a bigger molecule than the NP (its size depends on its molecular weight and shape). According to the Zetasizer results, the zeta potential of the nanoparticles increased, which is an indication of the binding of the enzymes to the surface of the nanoparticles. The application of larger amounts of enzymes and the attempt to saturate the surface of AuNPs would lead to a significant increase in the zeta potential that is undesirable because it would compromise the stability of the complex.

The activity of low amounts of enzyme in the complex was proved by the fact that no protein bands were observed in SDS-PAGE after washing the AuNP-enzyme complex, but the complex exhibited activity toward its substrates. Thus, the use of lower amounts of the enzyme in the AuNP-enzyme complex led to the desired result. The bands resulting from the degradation of these two proteins (fibrinogen and collagen type I) differ from pure proteinase K and AuNPs-proteinase K. The activity of proteinase K in the AuNP-proteinase K complex probably decreased because the amount of enzyme linked to AuNPs surface after the washing steps will also be lower than the starting amount (the starting amount corresponded to the tested concentration of pure enzyme). The adsorption of enzyme in AuNP-proteinase K complex is also documented by the shift in the complex surface charge in comparison to AuNPs (− 33 mV vs − 13 mV).

Importantly, the adsorption of proteinase K, DNase I, and RNase A onto the surface of the AuNPs did not change the three-dimensional structure of the enzymes, which is an important characteristic of promising candidates for enzyme delivery in anticancer therapy^3,22,52–54,59^. Next, the activity of enzymes was not affected by their adsorption onto AuNPs even though they underwent several washing steps and centrifugation. Even the complexes that possessed a potentially lower stability due to higher zeta potential exhibited digestion activity. It is important to note that the enzyme activity was different in each type of complex and the best enzymatic activity was found for AuNPs-proteinase K.

## Conclusion

This is the first report on the direct binding of various enzymes to biologically produced AuNPs. We showed that the enzymes (proteinase K, DNase I, and RNase A) adhere to the nanoparticles and retain their activity against various substrates. Remarkable degradations of dsDNA by AuNPs-DNase I and of protein substrates by AuNPs- proteinase K were observed. Those activities depended on the substrate and enzyme used. For example, the efficacy of DNase I was shown to be reduced by physical denaturation. Although we could not prove the hydrolysis of the capping agent after the addition of enzymes, the obtained data suggest that the enzymes were directly conjugated with AuNPs, which can be further used in cancer enzymotherapy.

## Methods

### Preparation and characterization of AuNPs before incubation with the enzymes

AuNPs were prepared using *F. oxysporum* (CCF 3732) culture supernatant. Cell-free supernatant was challenged with gold salt (HAuCl_4_.3H_2_O, AuNPs were then collected and washed three times as described in our previous study^29^. Formation of AuNPs was recognized by the color change of the sample (from yellow to deep red). AuNPs were collected, dispersed in nuclease-free ddH_2_O (Qiagen, Czech Republic), and used for subsequent tests. The characterization of the produced AuNPs consisted of visible spectrophotometry, TEM, energy dispersive X-ray spectroscopy (EDS), Fourier transform infrared spectroscopy (FTIR), zeta potential and size distribution (zeta sizer) analyses. We followed established protocols that were described earlier^28,29^.

### TEM and EDS

Size and composition of the nanoparticles were determined with a transmission electron microscopy (TEM, JEOL JEM -F 200) and an energy dispersive X-ray spectroscopy (EDS). An aliquot of 2 µL of the sample was placed on a glow discharge activated (30 s, 1 kV, 10 mA) grid. The samples were air-dried and analyzed using TEM. Specific areas of the TEM image were further analyzed using EDS. The instrument was operated at 200 kV and equipped with Cold FEG, TVIPS XF416 camera and JED 2300 X-ray spectrometer (JEOL, Freising, Germany).

### Graphite furnace atomic absorption spectroscopy (GF-AAS)

The amounts of ionic gold (Au^3+^) in three independent AuNP batches were analyzed using a GF-AAS instrument (Perkin Elmer AAnalyst 800, USA) in the presence of calibration standards containing 0, 1, 3, 10, 30, and 100 ng mL^−1^ Au. The instrument was operated with a hollow cathode Au lamp at 242.8 nm, a lamp current of 10 mA, a slit width of 0.7 nm (L), and a permanent Ir modification of the graphite furnace^60^. Freshly prepared aqua regia (concentrated HNO_3_ and HCl in a ratio of 1:3 (v/v) was used for sample preparation and the resolution of AuNPs^60^.

### FTIR

FTIR spectral analysis was then performed to determine the surface composition of the AuNPs.

The AuNPs were freeze-dried and the IR measurement was performed using a Vertex 70v FTIR spectrometer (Bruker Optics GmbH, Germany) equipped with a mercury cadmium telluride (MCT) detector. The sample was placed in a DC3 diamond compression cell (Specac Ltd., UK) and its IR absorption spectrum was recorded against a clean DC3 cell at wavenumbers of 4000–900 cm^−1^ in a nitrogen gas atmosphere. This method omits water vapor and CO_2_ peaks. The number of scans was 128–256 with a resolution of 4 cm^−1^. Spectrum was recorded and processed using OPUS software.

### Incubation of AuNPs with different enzymes

Produced AuNPs naturally coated with capping agents hypothetically containing nucleic acids and peptides/proteins were subjected to different enzymes. After the incubation of AuNPs with different enzymes and centrifugation of the samples, the resulting pelleted particles (AuNPs-enzymes, for the detection of adsorbed enzymes) and the resulting supernatants (for the detection of capping agent originated digestion products) were further tested with regard to possible enzymes adsorption as well as nucleic acid/peptide/protein content (digestion products), respectively.

### Capping agent digestion assessment

In order to test the digestion by DNase I, a RNase-free DNase I kit (Qiagen, Prague, Czech Republic) was used. According to the manufacturer’s instructions, the lyophilized enzyme was dissolved in 550 µL of nuclease-free ddH_2_O, 50 µL of the enzyme was added to 350 µL of RDD buffer, and the solution was added to 50 µL of AuNPs in water. The samples were incubated at room temperature (RT) for 15 min and centrifuged at 22,000*g* for 30 min. The supernatant was collected to analyze the nucleic acid content by gel electrophoresis and Nanodrop spectrophotometry (Thermo Fisher Scientific, USA). The pellet was washed three times (centrifuged at 22,000*g* for 30 min) in phosphate-buffered saline (PBS), and the vial was changed between each washing step to reduce the likelihood of the presence of adsorbed enzyme on the plastic. The pellet was dispersed in 50 µL RDD buffer, and stored at − 20 °C before enzyme adsorption assessment.

RNase A (Macherey-Nagel, Germany) was used for RNA digestion. According to the manufacturer’s instructions, the lyophilized enzyme was dissolved in 1000 µL of nuclease-free ddH_2_O, and 50 µL of the enzyme was added to 50 µL of AuNPs in water. The samples were incubated at RT for 15 min and centrifuged at 22,000*g* for 30 min. The supernatant was collected to analyze the nucleic acid content by gel electrophoresis and Nanodrop spectrophotometry. The pellet was washed three times in PBS (centrifuged at 22,000*g* for 30 min) and the vial was changed between each washing step. The pellet was dispersed in 50 µL of PBS and stored at − 20 °C before enzyme adsorption assessment.

Proteinase K (Macherey-Nagel, Germany) was used for protein digestion. The lyophilized enzyme was dissolved in proteinase buffer PB according to the manufacturer’s instructions. 50 µL of 20 mg/mL proteinase K was added to 500 µL of AuNPs. Incubation was performed at 37 °C for 1 h. The sample was divided into two vials, one of which was centrifuged at 22,000*g* for 30 min, the supernatant was collected, and the pellet was washed three times with PBS (the vial was changed between each washing step) to analyze the digestion product and enzyme adsorption, respectively, and the other was stored unwashed at − 20 °C until further experiments. All vials were subjected to SDS-PAGE analysis to visualize the digestion products and AuNPs-enzyme.

In order to check the activity of the pure enzymes, various RNA, DNA, and protein samples served as digestion substrates. A ssRNA oligonucleotide 5′ rUrCrA rCrArU rArGrG rArArU rGrArA rArArG rCrCrA rUrA—3′ was used as substrate for RNAse A. ssDNA oligonucleotide 5′ ACC CTT CAC CAA TGA CTC CTA TG 3′ and genomic dsDNA (from BALB/c female mouse) were used as substrates for DNAse I. The initial concentration of each nucleic acid is listed in Table 2. To check the digestion of nucleic acids, aliquots of ssRNA, ssDNA, and dsDNA solutions were subjected to the same enzymatic digestion as described above. Successful digestions of those samples proved the correct enzymatic activities of DNase I and RNase A enzymes.

Two different proteins, collagen type I solution from rat tail (4 mg/mL, Sigma-Aldrich, Prague, Czech Republic) and fibrinogen (2 mg/mL, Sigma-Aldrich, Prague, Czech Republic) were used as substrates of proteinase K and were subjected to the same enzymatic digestion as described above.

### Evaluation of the adsorption of the enzymes onto the AuNPs

To test the adsorption hypothesis, AuNPs-enzymes (i.e., the pellets from the previous step in which digestion of the capping agent was evaluated) were considered as test samples (assumed to be active enzyme adhered to AuNPs) and equal amounts of the DNA and RNA samples were digested by those AuNP suspensions following the same protocol as described above. Briefly, for DNA digestion, 10 µL of each of the enzymes (DNase I and AuNPs-DNase I as control and test, respectively) were added to 70 µL of RDD buffer, and the solutions were added to 10 µL of ssDNA or dsDNA. The samples were incubated at RT for 15 min. For RNA digestion, 10 µL of each of the enzymes (RNase A and AuNPs-RNase A as control and assay, respectively) were added to 10 µL of ssRNA and the samples were incubated at RT for 15 min. For protein digestion, collagen type I and fibrinogen were digested with proteinase K and AuNPs- proteinase K as the control and test, respectively. The protocol was the same as described above.

### Characterization of AuNPs after incubations with the enzymes

After the application of different enzymes, the AuNPs were characterized by spectrophotometer and Zetasizer.

### Visible spectrophotometry

The maximum absorbance peaks of the washed AuNP samples after incubation with enzymes in the presence of AuNPs as a control were determined using a Tecan spectrophotometer (Infinite 200 PRO, Männedorf, Switzerland) and ddH_2_O served as a blank. The wavelengths used were between 400 and 700 nm^29^.

### Zeta potential and size analysis

A Zetasizer Ultra (Malvern Panalytical, UK) was used and the size and zeta potential were determined according to our previous study. The ZEN2112 quartz cuvette and the DTS1070 zeta cell with folded capillaries were used to determine the size and zeta potential of AuNPs, both for the control AuNPs and for washed AuNP samples after incubation with enzymes^28^.

### Nucleic acid and protein electrophoresis

Electrophoresis was employed to: (1) visualize products (nucleic acids and proteins) resulting from the digestion of capping agents, (2) visualize the activities of pure enzymes (as controls) on different substrates as described above, and (3) visualize the activities of AuNPs-enzymes (as assays) on different substrates. In agarose gel electrophoresis, the intact macromolecules were used as controls. In SDS-PAGE analysis, the original enzyme without AuNPs, the AuNPs-enzyme (before and after washing), and the intact substrates were used as controls.

For DNA and RNA agarose gel electrophoresis, 2% agarose (Sigma Aldrich, Prague, Czech Republic) containing GelRed (Biotium, Czech Republic) was prepared in Tris-acetate-EDTA buffer (TAE) and 3 µL of 6X MassRuler DNA loading dye (Thermo Fisher Scientific, USA) was mixed with 15 µL of each sample. Electrophoresis was performed at 110 V, 20 W, 150 mA for 40 min in the presence of MassRuler DNA Ladder (Thermo Fisher Scientific, Prague, Czech Republic) and the gel was visualized under a UV transilluminator. The nucleic acid content of the digested DNA and RNA molecules and the undigested control molecules was determined with a Nanodrop spectrophotometer.

To use SDS-PAGE for the detection of protein digestion, 6.25 µL of 4X NuPAGE LDS Sample Buffer (Thermo Fisher Scientific, Prague, Czech Republic) was mixed with 18.75 µL of the samples and incubated at 100 °C for 10 min. Samples were cooled and electrophoresed in a 1D NuPAGE 10% Bis–Tris gel with a 1.0 mm × 10 well (Invitrogen, Thermo Fisher Scientific, Prague, Czech Republic) in the presence of PageRuler Prestained Protein Ladder (Thermo Fisher Scientific, Prague, Czech Republic). The gel was run in 1X NuPAGE MOPS SDS running buffer (Thermo Fisher Scientific, Prague, Czech Republic) in an XCell SureLock™ Mini-Cell Tank (Thermo Fisher Scientific, Prague, Czech Republic) at 100 V, 150 mA, 100 W for 30 min. Bands were stained with SimplyBlue SafeStain (Thermo Fisher Scientific, Prague, Czech Republic) and visualized after washing with ddH_2_O for 1 h.

### Liquid chromatography–mass spectrometry (LC–MS) analysis

To demonstrate the binding of the enzymes to the surface of the AuNPs, LC–MS analysis of the samples was performed, with each AuNPs-enzyme sample prepared in three replicates on three different days. The sample preparation protocol for LC–MS analysis and the protocol used for this experiment are the same as in our previous work^29^. Results were compared against the UniProt database for all taxa.

## Data Availability

The data supporting the results of the current study are available in the article file. All other data are available from the corresponding author upon request.
